# Carbohydrate-Protein drink is effective for restoring endurance capacity in masters class athletes after a two-Hour recovery

**DOI:** 10.1080/15502783.2023.2178858

**Published:** 2023-02-26

**Authors:** Erica R. Goldstein, Jeffrey R. Stout, Adam J. Wells, Jose Antonio, Ecaterina Vasenina, David H. Fukuda

**Affiliations:** a Department of Health Sciences, Stetson University, Deland, Florida, USA; b Physiology of Work and Exercise Response (POWER) Laboratory, Institute of Exercise Physiology and Rehabilitation Science, University of Central Florida, Orlando, Florida, USA; c Exercise Physiology Intervention and Collaboration (EPIC) Laboratory, Institute of Exercise Physiology and Rehabilitation Science, University of Central Florida, Orlando, Florida, USA; d Department of Health and Human Performance, Fight Science Laboratory, Nova Southeastern University, Davie, FL, USA

**Keywords:** Protein, carbohydrate, post-exercise, fatigue, glycogen, recovery

## Abstract

**Background:**

Carbohydrate (CHO) and carbohydrate-protein co-ingestion (CHO-P) have been shown to be equally effective for enhancing glycogen resynthesis and subsequent same-day performance when CHO intake is suboptimal (≤0.8 g/kg). Few studies have specifically examined the effect of isocaloric CHO vs CHO-P consumption on subsequent high-intensity aerobic performance with limited time to recover (≤2 hours) in masters class endurance athletes.

**Methods:**

This was a randomized, double-blind between-subject design. Twenty-two male masters class endurance athletes (age 49.1 ± 6.9 years; height 175.8 ± 4.8 cm; body mass 80.7 ± 8.6 kg; body fat (%) 19.1 ± 5.8; VO_2peak_ 48.6 ± 6.7 ml·kg·min^−1^) were assigned to consume one of three beverages during a 2-hour recovery period: Placebo (PLA; electrolytes and water), CHO (1.2 g/kg bm), or CHO-P (0.8 g/kg bm CHO + 0.4 g/kg bm PRO). All beverages were standardized to one liter (~32 oz.) of total fluid volume regardless of the treatment group. During Visit #1, participants completed graded exercise testing on a cycle ergometer to determine VO_2_peak and peak power output (PPO, watts). Visit #2 consisted of familiarization with the high-intensity protocol including 5 × 4 min intervals at 70-80% of PPO with 2 min of active recovery at 50 W, followed by a time to exhaustion (TTE) test at 90% PPO. During Visit#3, the same high-intensity interval protocol with TTE was conducted pre-and post-beverage consumption.

**Results:**

A one-way ANCOVA indicated a significant difference among the group means for the posttest TTE (F_2,18_ = 6.702, *p* = .007, ƞ^2^ = .427) values after adjusting for the pretest differences. TTE performance in the second exercise bout improved for the CHO (295.48 ± 24.90) and CHO-P (255.08 ± 25.07 sec) groups. The water and electrolyte solution was not effective in restoring TTE performance in the PLA group (171.13 ± 23.71 sec).

**Conclusions:**

Both CHO and CHO-P effectively promoted an increase in TTE performance with limited time to recover in this sample of masters class endurance athletes. Water and electrolytes alone were not effective for restoring endurance capacity during the second bout of exhaustive exercise.

## 1. Background

The rate of participation by masters class athletes (MCAs) in organized endurance and ultra-endurance events (>6 hours) has increased exponentially over the past 10–15 years [1,2]. MCAs are individuals generally classified as ≥ 35 years of age who meticulously train for and compete in organized sports [2–5]. Carbohydrate and protein are key nutritional factors that promote glycogen and protein synthesis, support recovery, and allow for repetitive high-intensity efforts. Reaburn et al. (2015) thought that based on the available science, MCAs should take a whey protein supplement (combined with CHO) right after exercise to promote rapid recovery [6]. Few studies, however, have examined short-term (2–6 hours) post-exercise recovery nutrition specific to MCAs [7,8].

Two different isoenergetic options have been shown to effectively promote glycogen resynthesis post-exercise: CHO (1.2 g/kg) and CHO-P (0.8 g/kg CHO + 0.4 g/kg PRO) [9–11]. CHO-P is not substantially more useful for promoting glycogen resynthesis when protein is added to CHO in the amount of 1.2 g/kg [12–14]. Therefore, CHO availability refers to the consumption of CHO in the amount of approximately 1.2 g/kg bm or the addition of protein during the recovery period when CHO intake is insufficient (i.e. ≤ 0.8 g/kg) and/or time is limited, and athletes are unable to match nutrient demand with intake.

Masters class endurance athletes frequently train in the evening and the next morning, on successive weekend days, or twice per day one-to-two times per week when preparing for a triathlon. A high weekly training volume combined with the demands of family and professional responsibilities is a considerable time investment [15,16]. Therefore, nutrient intake during the immediate post-exercise recovery period is essential and insufficient nutrient intake due to time constraints may compromise the ongoing recovery of muscle and glycogen stores, along with subsequent performance [17]. In support, a recent investigation reported time to exhaustion performance increased by approximately 35% during the second bout of exercise in college-aged males and females following consumption of a CHO-P (0.8 g/kg and 0.4 g/kg) beverage and a 2-hr recovery period [18]. In comparison, performance decreased by 14% and 31% with CHO (1.2 g/kg) and PLA (electrolytes and water), respectively [18]. Dahl et al. (2020) reported endurance athletes (26.7 ± 1.7 years) were able to cycle 8.4 ± 1.8 min longer following a 5-hour recovery period, in favor of CHO-P as compared to an isoenergetic CHO beverage, despite similar glycogen synthesis between groups [11] [19]. These and other studies have shown that CHO-P was associated with either an increase in performance or an attenuated decrement in performance during a subsequent bout of endurance exercise and may be a viable option for promoting recovery when the time period between bouts of exercise is limited [11,18,19].

Protein intake promotes skeletal muscle remodeling and repair, adaptation to training, and replenishment of energy stores [20,21]. Therefore, post-exercise protein consumption should be of equal priority to that of CHO. Formative studies aimed at determining the appropriate amount of protein to support muscle protein synthesis have centered around an absolute dose of 20 g of protein, in response to resistance exercise, and in young men [22–24]. Moore et al. (2015) suggested that relative to body mass, healthy older men require 0.40 g/kg of protein to maximally stimulate muscle protein synthesis, whereas young men require 0.24 g/kg [25]. Similarly, Moore (2021) has recommended master endurance athletes consume 0.5 g/kg of a rapidly digested leucine-rich protein meal within the immediate recovery period to promote muscle protein synthesis [26].

A post-exercise and (daily) per meal intake of 0.40 g/kg of protein or approximately 30 g has been recommended by others to promote the repair and remodeling of skeletal muscle in master athletes who regularly engage in endurance exercise [21,27].

Exercise-induced dehydration can impair thermoregulation and promote cardiovascular strain during a bout of prolonged endurance exercise [28]. Both heart rate variability and heart rate recovery (HRR) have been used as indicators of cardiac autonomic function during recovery from exercise [29]. Some studies have specifically examined the effect of consuming water or an isotonic solution on parasympathetic reactivation upon exercise cessation [30–32]. HRR is a valid and simple measure that is easily calculated at the cessation of exercise as the difference between peak heart rate (beats per min) and the heart rate 1-, 2-, 3-, and 5-min later [33]. Heart rate recovery index (HRRi), a novel index adapted from the combat sports literature [34], accounts for total work (i.e. HRR relative to work) and can be useful in exercise trials where total work varies between participants, such as with time to exhaustion. To our knowledge the effect of isocaloric recovery drinks on HRRi in male MCAs has not been examined.

While there is an increased rate of participation by MCAs in endurance sports, a critical gap in the research exists related to post-exercise nutrition specific to MCAs and short-term (2 hours or less) recovery. This is problematic as slower recovery rates between subsequent bouts of exercise have been demonstrated, and higher protein needs post-exercise have been indicated to promote muscle remodeling and repair in MCAs [7,8]. A unique opportunity exists to examine CHO-P in a 2-hour recovery period to improve the rate of recovery from prior exercise, support adaptation, and thus performance in a subsequent bout of training. Therefore, the primary purpose of this study is to examine the effects of CHO and CHO-P and placebo within a 2-hour recovery period on subsequent high-intensity exercise performance in MCAs.

## 2. Methods

### 2.1. Study design

**Figure 1.**
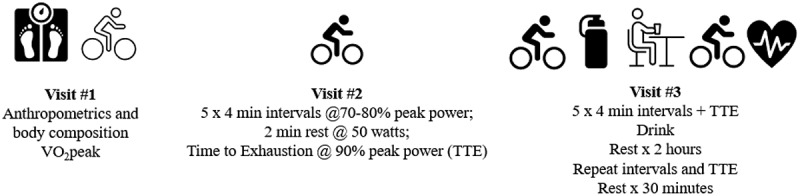
Timeline and exercise testing protocol.

This study utilized a randomized, double-blind, placebo-controlled between-subject design to examine the effects of CHO and CHO-P supplementation on short-term recovery following aerobic interval exercise and time to exhaustion testing in male MCAs. The athletes were required to visit the laboratory on three separate occasions, with testing visits separated by a minimum of 48 hours and completed within two weeks. Figure 1 outlines the timeline and exercise testing protocol. During the first visit, participants were assessed for height, weight, and body composition and then performed a graded exercise test on a cycle ergometer to determine VO_2_peak and peak power output (PPO; watts). Participants returned to the laboratory for a second time to become familiarized with the high-intensity aerobic interval protocol that consisted of 5 × 4 min intervals (INT) at 70–80% PPO followed by a time to exhaustion (TTE) test at 90% PPO [35]. Participants were randomized to receive one of three recovery beverages that were consumed during a 2-hr recovery period. During the third and final visit participants performed the INT and TTE testing pre-post beverage consumption. All three recovery beverages were matched for a total fluid volume of 32 oz or approximately 1 liter and the CHO (1.2 g/kg bm) and CHO-P (0.8 g/kg bm CHO + 0.4 g/kg PRO) drinks were matched for calories. Participants were weighed in their cycling bib shorts immediately before and after the first and second bouts of exercise and fluid loss was determined based on the difference between pre- and post-weight changes for each bout of exercise [36]. To assess HRR, participants were instructed to keep the heart rate monitor in place and then rested in a seated position for an additional 30 min after the second bout of INT and TTE testing. Participants were asked to keep dietary intake consistent on the day of familiarization and the experimental protocol and provided a dietary recall typical of days that they train. The familiarization and the experimental protocol occurred at approximately the same time of day for each participant. Participants were instructed to avoid exercise for 24 hours prior to the final day of testing (experimental protocol).

### 2.2. Subjects

**Figure 2.**
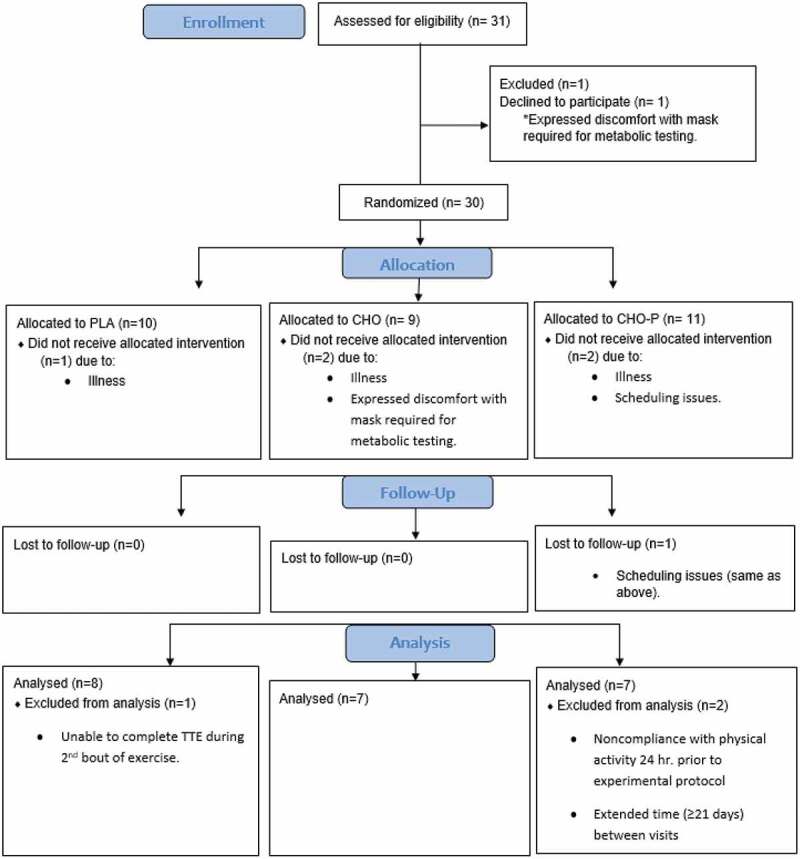
Consort flow diagram.

Twenty-two male MCAs (49 ± 6 years, VO_2peak_ 48.6 ± 6.7 mL·kg·min^−1^) completed this research study (Table 1). This investigation was approved by the university’s Institutional Review Board (STUDY00002618) and listed on ClinicalTrials.gov (NCT04859491; 3/30/21). The calculation of sample size (G*Power 3.1.9.4, Dusseldorf, Germany) was based on pilot data in moderately trained young men and women (22.0 + 2.4 years) collected in our lab using the same protocol (90% PPO TTE) as outlined in this study [18]. The sample size was determined to be a total of 18 (6 in each group) based on a one-way ANCOVA, power of 0.80, and an alpha of 0.05. After participants signed the informed consent, they completed the American College of Sports Medicine Exercise Preparticipation Health Screening Questionnaire for Exercise Professionals (PHSQEP), Physical Activity Readiness Questionnaire for Everyone (PAR-Q+), and Medical Health and Activity Questionnaire (MHAQ). Participants were excluded if they had any recent musculoskeletal injuries or surgeries, cardiovascular or metabolic disease, or any chronic illness that required continuous medical care. Full CONSORT information is reported in Figure 2.

**Table 1.** Mean and SD values for demographic data.

	Total (n = 22)	PLA (n = 8)	CHO (n = 7)	CHO-P (n = 7)
Age (years)	49.1 ± 6.9	51.8 ± 4.7	44.1 ± 8.4	51.0 ± 5.3
Height (centimeters)	175.8 ± 4.8	175.2 ± 5.9	177.8 ± 4.4	174.5 ± 3.6
Weight (kilograms)	80.7 ± 8.6	78.0 ± 8.0	80.9 ± 9.2	83.5 ± 9.1
Body fat (%)	19.1 ± 5.8	20.6 ± 5.7	19.4 ± 3.3	16.6 ± 8.2
Fat-Free Mass (kilograms)	64.8 ± 6.0	61.8 ± 6.8	64.7 ± 5.6	68.8 ± 2.9
VO_2_peak (mL•kg^−1^•min^−1^)	48.6 ± 6.7	46.2 ± 3.5	53.3 ± 6.3	46.8 ± 8.2
Peak Power Output (watts)	291.8 ± 39.5	277.5 ± 31.1	308.6 ± 37.6	291.4 ± 48.1
Cycling (hr/wk)	7.7 ± 2.0	7.7 ± 1.7	8.0 ± 1.6	7.4 ± 2.6

Cycling was the primary sport reported by 18 participants: PLA (n = 7), CHO (n = 4), CHO-P (n = 7). Body fat (%) and fat-free mass was reported for all but one participant in the CHO-P group.

### 2.3. Procedures

*Anthropometrics and Body Composition*. Height and body weight were assessed using a stadiometer and scale (Health-o-meter Professional Patient Weighing Scale, Model 500 KL, Pelstar, Alsip, IL, USA), and body composition was assessed using bio-electrical impedance spectroscopy analysis (SOZO, Carlsbad, CA, USA). Participants were tested barefoot wearing minimal clothing.

*Graded Exercise Test and Indirect Calorimetry*. All participants performed a graded exercise test (GXT) to volitional exhaustion on a cycle ergometer (Lode, Corival cpet, Groningen, The Netherlands) to determine VO_2peak_ and PPO. Prior to testing, each participant was fitted with a Polar heart rate monitor (chest strap and sensor; Polar H10, Polar Electro Oy, Kempele, Finland) to record heart rate. Seat height was set based on feedback from each participant and then recorded and kept constant throughout the study. Participants then completed a five-minute warm-up on the cycle ergometer at a self-selected intensity and cadence. The test consisted of 2-minute stages, beginning at an initial workload of 50 watts (W), then 100 W, then 150 W followed by an increase of 30 W every 2 minutes until the participant could no longer maintain 60 revolutions per minute [35]. Open-circuit spirometry was used to estimate VO_2_peak (L·min^−1^) by sampling and analyzing the breath-by-breath expired gases with a calibrated metabolic cart (True One 2400® Metabolic Measurement System, Parvo-Medics Inc., Sandy, UT). Ventilation and expired gases were continuously recorded and averaged every 15s to determine VO_2_peak, which was defined as the highest peak value achieved during the last completed stage of the test coinciding with at least two of the following three parameters: heart rate (HR) within 10% of age-predicted maximal HR; respiratory exchange ratio (RER) of 1.15 or higher; a plateau in oxygen consumption despite an increase in exercise intensity. The highest power output achieved during the last completed 2-min stage was recorded as PPO in watts [18].

*High-Intensity Aerobic Interval and Time to Exhaustion Protocol*. Participants performed a warm-up on a cycle ergometer that began with 3-minutes at 50 W, 2-minutes at 100 W, and 1-minute at 75 W [35]. Participants then performed 5 x 4-minute high-intensity aerobic intervals at 70–80% of individual PPO, as determined by the familiarization trial. During the familiarization trial, each person attempted to complete all five intervals at 80% of individual PPO immediately followed by the TTE trial at 90% of PPO. If a participant was unable to complete the intervals at 80% of individual PPO or was able to complete the intervals but not the TTE trial, then the power was decreased by 5% (to a minimum of 70% PPO) until the participant could perform the entire series of aerobic intervals and TTE protocol [18]. Two-minutes of low-intensity cycling at 50 W separated each aerobic interval. Immediately following the fifth interval, participants cycled at a work rate corresponding to 90% PPO until volitional exhaustion or when rpm fell below 60 for 10 seconds. Following the endurance trial (INT and TTE), participants cycled with no resistance for 5 minutes to cool down. Revolutions per minute were the only performance measure visible to participants, and verbal encouragement was provided throughout the aerobic intervals and time to exhaustion. Rating of perceived exertion (RPE) was recorded using the Borg 10 scale during the last 30 seconds of each aerobic and active rest interval. Gas exchange data was collected with a metabolic analyzer (Trueone 2400, Parvo Medics, Utah, USA) throughout the experimental protocol for both bouts of INT and TTE testing.

*Supplementation*. Participants were randomly assigned via a web-based random assignment generator (Research Randomizer; www.randomizer.org) to receive one of three beverages during a 2-hour recovery period. Participants consumed either placebo (PLA; Gatorade® Zero Thirst Quencher Orange, Chicago, IL, USA), CHO (6% carbohydrate-electrolyte solution; Gatorade® Thirst Quencher Orange, Chicago, IL, USA) in the amount of 1.2 g/kg of body weight, or CHO with whey protein isolate powder (CHO-P; 6% carbohydrate-electrolyte solution Gatorade® Thirst Quencher Orange, Chicago, IL, USA; BiPro Elite, Agropur Inc., Appleton, WI, USA) in the amount of 0.8 g/kg of body weight and 0.4 g/kg of body weight, respectively. If necessary, Gatorade® Thirst Quencher Orange powder (Chicago, IL, USA) was weighed in grams via a digital food scale and added to the existing liter of fluid to reach the total amount of CHO needed. Fat-free mass (kg) was used to calculate the CHO or CHO-P needs for any participant with a measured body fat percentage that was greater than or equal to 20%. The total fluid volume of each recovery drink regardless of treatment was 32 ounces or approximately 1 liter. Participants were allowed sips of water as needed during the 2-hr recovery period. The 32 ounces of fluid provided as part of the treatment in addition to sips of water were recorded for each participant as total fluid consumed during the 2-hour recovery period. Participants were instructed to consume the recovery beverage ad libitum but in its entirety within 90 min of completing the first bout of exercise. To disguise appearance, all three recovery beverages were prepared in the same dark red Nalgene water bottles (Nalge Nunc International Corporation, Rochester, NY, USA). The consistent orange flavor and the opacity of the bottle resulted in effective blinding to the participant. In a pilot study done in our lab using a between-subject design, there were approximately 42.1% correct guesses when participants consumed all three recovery beverages.

*Heart Rate Recovery*. Heart rate (beats per minute; bpm) was recorded via the heart rate monitor and Elite HRV® smartphone app. Heart rate was continuously recorded every 3 s and downloaded using a laptop with commercially available heart rate variability analysis software (Kempele, Finland; Kubios HRV Analysis v 3.3, Kuopio, Finland). HRRi was calculated as the last 3-sec peak heart rate value (bpm) post-exercise plus the heart rate (bpm) recorded 1 min post-exercise, divided by total work. The HRRi was adapted from a previously published formula used by others [34]. Total work was calculated as TTE in sec x 90% PPO divided by 1000 (kilojoules). HRRi was calculated 1-, 2-, and 5-min post-exercise. A smaller HRRi value indicated improved recovery.

$$HRRi=\frac{Final\;peak\;heart\;rate\;\left(bpm\right)\;+\;Heart\;Rate1min\;\left(bpm\right)}{TTE\;\left(sec\right)\;x\;90\%\;PPO}$$

*Nutrient Intake and Dietary Recall*. Participants were asked to complete the ASA24® during the 5- to 9-day period between familiarization and the experimental protocol. The ASA24® is a validated, automated self-administered 24-hour dietary assessment tool developed by the National Cancer Institute (Bethesda, Maryland) [37]. Participants were emailed detailed instructions on how to complete the electronic diet recall and an individual username and password to access the ASA24® system. During the recall, participants received automated prompts that would assist them in quantifying portion sizes, the actual volume of food consumed at each meal or snack, and commonly forgotten items (condiments, supplements, sugar-sweetened beverages) [38]. Participants were asked to report a 24-hour intake that is typical for days that they train. The ASA24® dietary recall assessment was utilized to estimate the mean total energy intake in kilocalories (Kcal).

*Statistical Analysis*. All statistical analyses were conducted via the Statistical Package for Social Science (SPSS) software for Windows version 28 (SPSS Inc., Chicago, IL). Descriptive statistics were calculated to determine group demographics. Before analysis, all data was tested for normality (Shapiro-Wilks), homogeneity of variance (Levene’s test of equality of error variance), and homogeneity of slopes. Data was statistically analyzed using a separate 1-way analysis of covariance (ANCOVA) for TTE and HRRi. The pretest and the posttest values were used as covariate and dependent variables, respectively. If ANCOVA assumptions, such as homogeneity of slopes, were not met, then Quade’s nonparametric analysis was used. Differences in total energy intake, macronutrients, and fluid loss were assessed with a one-way ANOVA. Fisher’s LSD Post-Hoc analysis was used to determine group differences. For effect size (ES), the partial eta squared statistic was reported, with 0.01, 0.06, and 0.14 representing small, medium, and large ES, respectively [39]. The significance level was set at p ≤ 0.05.

## 3. Results

### 3.1. Participants

Data from 22 trained male MCAs (49 ± 6 years old) were used in this research investigation. Thirty-one athletes were recruited; however, three were withdrawn from the study due to illness (i.e. COVID, flu); two discontinued participation after expressing discomfort with the mask required for metabolic testing; and one was withdrawn due to scheduling issues (Figure 2). Data for three athletes were omitted from analysis due to the following: noncompliance with activity 24 hours prior to the experimental protocol, inability to complete TTE testing following the second bout of aerobic intervals, and extended time between visits (i.e. ≥ 21 days). The MCAs in this study met the eligibility criteria of a minimum of three years participating in endurance sports. Nineteen participants reported engaging in regular endurance activity: 18 participants reported cycling 7.7 ± 2.0 hours per week, six reported running 2.5 ± 2.3 hours per week, and two participants reported swimming 2.0 ± 1.4 hours per week. The average VO_2_peak was 48.6 ± 6.7 mL·kg·min^−1^. Nine of 22 total participants had a body fat percentage greater than 20% with three being placed into PLA group; therefore, fat-free mass (kg) was used to calculate the nutrient needs of four (18.1%; three placed in the CHO group and one in the CHO-P group) participants.

### 3.2. Blinding

Blinding results were available for 19 of 22 total participants with 31.6% (n = 6) correctly identifying which treatment beverage they consumed.

### 3.3. High-Intensity Aerobic Interval and Time to Exhaustion

**Figure 3.**
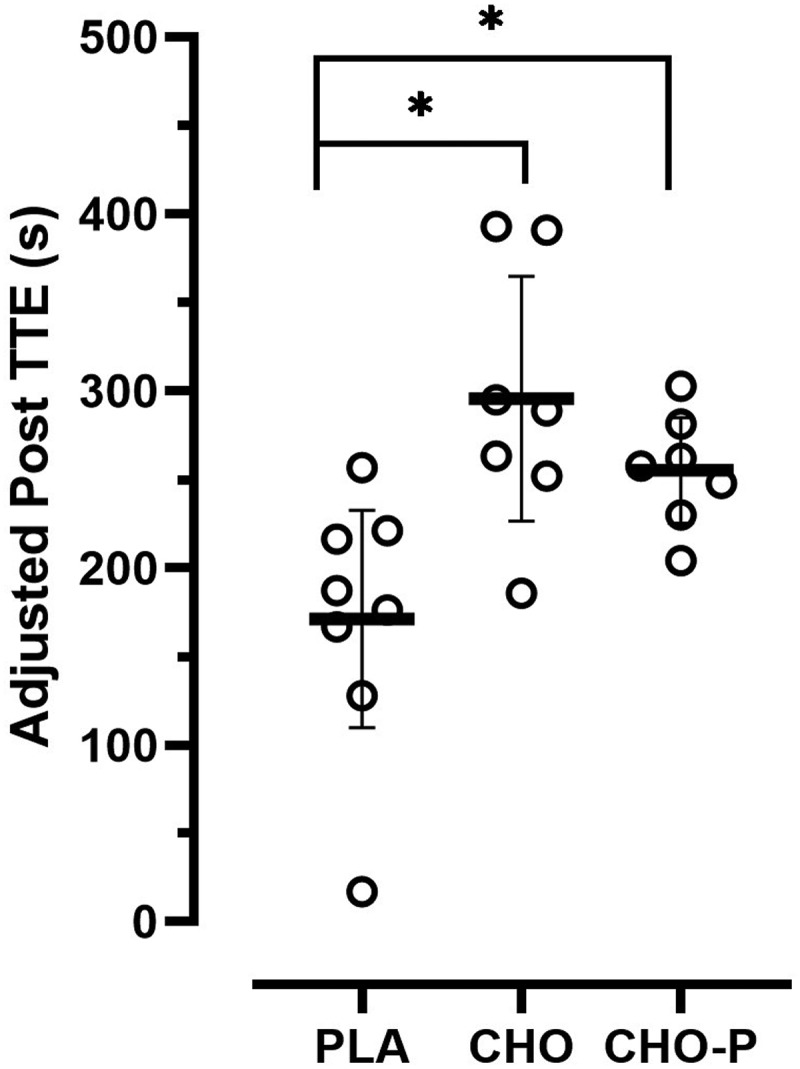
Group mean values (±SE) for posttest time to exhaustion scores adjusted for the initial differences in the pretest time to exhaustion (covariate). The adjusted pretest value was 203.05 sec. *Indicates significant difference compared to PLA.

Table 2 shows the group means (±SD) for the pretest and posttest values. Figure 3 shows the group means (±SE) for the posttest TTE (sec) values adjusted for the initial differences in the pretest TTE. There was homogeneity of regression slopes as the interaction term was not statistically significant (F_2,16_ = 2.756, *p* = .094). Assumptions were met for normality and there was homogeneity of variance for the TTE values. The ANCOVA indicated a significant difference (F_2,18_ = 6.702, *p* = .007, ƞ^2^ = .427) among the group means for the posttest TTE values after adjusting for the pretest differences. The strength of the association (i.e. effect size, ƞ^2^) indicated that the treatment groups (PLA, CHO, and CHO-P) accounted for 43% of the variance of the posttest TTE values. Fisher’s LSD pairwise comparisons indicated that the posttest TTE was greater in CHO (*p* < .002) and CHO-P (*p* = .028) when compared to the PLA group with no differences between CHO and CHO-P (*p* = .265).

**Table 2.** Mean and SD values for time to exhaustion (sec) at pretesting and posttesting for each group.

TTE		PLA (n = 8)	CHO (n = 7)	CHO-P (n = 7)
Pretest	Mean	165.00	218.57	231.00
	SD	79.82	138.70	133.60
Posttest	Mean	132.75	311.14	283.29
	SD	64.28	167.90	158.90

### 3.4. Heart rate recovery

Table 3 shows the group means (±SD) for the pretest and posttest HRRi values at three separate time points. The assumption of normality and equality of variance was not met; therefore, the Quade nonparametric ANCOVA was used and indicated a significant difference among the group means for the posttesting HRRi values at 1-min (F_2,17_ = 6.715, p = .007), 2-min (F_2,17_ = 5.720, p = .013), and 5-min (F_2,17_ = 6.438, p = .008). Fisher’s LSD pairwise comparisons indicated the posttest HRRi was significantly different between PLA and CHO at 1-min (p = .003), 2-min (p = .005), and 5-min (p = .003) posttesting. Post hoc testing indicated the posttest HRRi was significantly different between PLA and CHO-P at 1-min (p = .026), 2-min (p = .029), and 5-min (p = .025) posttesting. There were no differences in posttest HRRi values between the CHO and CHO-P groups at any of the three posttesting time points: 1-min (p = .469), 2-min (p = .628), 5-min (p = .549).

**Table 3.** Mean and SD values for heart rate recovery index at pretesting and posttesting for each group.

HRRi		PLA (n = 8)	CHO (n = 7)	CHO-P (n = 5)
	**1-min**			
Pretest	Mean	9.77	6.95	8.26
	SD	5.18	3.0	8.33
Posttest	Mean	13.54*†	5.10	7.66
	SD	10.85	2.84	9.10
Delta Score (Post-Pre)	Mean	3.77	−1.86	−0.59
	SD	8.37	1.99	1.01
	**2-min**			
Pretest	Mean	9.13	6.64	7.86
	SD	4.80	2.93	8.05
Posttest	Mean	12.73*†	4.75	7.26
	SD	10.10	2.66	8.65
Delta Score (Post-Pre)	Mean	3.60	−1.89	−0.61
	SD	7.60	1.93	0.94
	**5-min**			
Pretest	Mean	8.84	6.43	7.63
	SD	4.70	2.81	7.93
Posttest	Mean	12.39*†	4.59	7.08
	SD	10.10	2.57	8.60
Delta Score (Post-Pre)	Mean	3.55	−1.84	−0.55
	SD	7.64	1.99	1.00

*Significant difference between PLA and CHO groups. †Significant difference between PLA and CHO-P groups.

### 3.5. Dietary analysis

Table 4 contains the mean and standard deviation values for total energy intake (kilocalories), protein, fat, and carbohydrate among the three groups (PLA, CHO, CHO-P) of participants. Due to compliance issues, data is only reported for 59% (13 of 22) of the participants: 5 of 8 participants in the PLA group; 4 of 7 participants in the CHO group; 4 of 7 participants in the CHO-P group. Assumptions were met for normality and there was homogeneity of variance for total calories, protein, fat, and carbohydrate as assessed by Levene’s test for equality of variances. A one-way ANOVA revealed no significant differences (*p* > .05) between total energy intake, protein, fat, or carbohydrate.

**Table 4.** Total energy intake and macronutrient values are reported for 13 of 22 participants. Values are expressed as mean ± standard deviation.

	Total (n = 13)	PLA (n = 6)	CHO (n = 4)	CHO-P (n = 4)
Weight (kilograms)	80.0 ± 7.7	77.6 ± 5.4	76.7 ± 2.9	86.3 ± 10.6
Total energy intake (kcal)	3011.9 ± 1063.2	2788.9 ± 1153.2	2916.2 ± 1123.7	3386.2 ± 1097.2
Total energy intake (kcal) (g/kg)	37.6 ± 12.9	36.1 ± 15.0	37.8 ± 13.6	39.4 ± 13.2
Protein (g)	142.7 ± 51.1	138.3 ± 65.6	136.8 ± 34.8	154.0 ± 57.0
Protein (g/kg)	1.8 ± 0.6	1.8 ± 0.9	1.8 ± 0.4	1.8 ± 0.7
Fat (g)	119.3 ± 49.1	105.6 ± 48.4	121.7 ± 66.6	134.0 ± 38.3
Fat (g/kg)	1.5 ± 0.6	1.4 ± 0.6	1.6 ± 0.8	1.6 ± 0.4
Carbohydrate (g)	338.5 ± 122.7	319.3 ± 135.8	333.7 ± 117.1	367.3 ± 142.0
Carbohydrate (g/kg)	4.3 ± 1.6	4.2 ± 1.8	4.3 ± 1.4	4.3 ± 1.8

### 3.6. Fluid loss

Table 5 contains the mean and standard deviation values for participant body mass (kg) measured at the start of and immediately following the first bout of exercise (i.e. INT and TTE), and at the conclusion of the 2-hour recovery period at the start of the second bout of exercise (INT and TTE). The assumption of normality was met for the PLA and CHO-P groups. The Shapiro-Wilk test indicated a significant departure from normality for the CHO group at each of the three time points (W = .705, *p* = .004, W = .710, *p* = .005, W = .700, *p* = .004). There was homogeneity of variance at the start of bout 1 (*p* = .967), immediately following bout 1 (*p* = .965), and at the start of bout 2 (*p* = .965). The deviation from normality for the CHO group was reviewed, and the one-way ANOVA was conducted. Results indicated no significant difference (*p* > .05) in participant body mass (kg) as measured at the beginning and end of the first bout of exercise, and at the start of the second bout of exercise.

**Table 5.** Participant fluid loss pre-post exercise and total volume consumed during the 2-hour recovery period. Values are expressed as mean ± standard deviation.

	Total (n = 22)	PLA (n = 8)	CHO (n = 7)	CHO-P (n = 7)
Exercise Bout 1: *Pre*-bike weight (kilograms)	80.7 ± 8.8	78.5 ± 7.7	80.5 ± 10.2	83.5 ± 8.9
Exercise Bout 1: *Post*-bike weight (kilograms)	80.1 ± 8.6	77.8 ± 7.6	79.8 ± 10.1	82.8 ± 8.7
Total Weight Loss (kilograms) *Bout 1*	0.7 ± 0.2	0.7 ± 0.2	0.7 ± 0.2	0.7 ± 0.3
Exercise Bout 2: *Pre*-bike weight (kilograms)	80.6 ± 8.6	78.3 ± 7.5	80.4 ± 9.9	83.5 ± 8.8
Volume of Fluid Consumed: Treatment beverage + sips of water (liters)	1.1 ± 0.2	1.0 ± 0.1	1.1 ± 0.3	1.1 ± 0.2

## 4. Discussion

The purpose of this investigation was to examine the effectiveness of three different beverages (CHO, CHO-P, and PLA) on short-term recovery from repeated bouts of exhaustive exercise in male endurance MCAs. The results support our hypothesis that CHO-P (0.8 g/kg bm CHO + 0.4 g/kg bm PRO) was equivalent to CHO (1.2 g/kg bm) for promoting an increase in time to exhaustion (TTE) performance following a 2-hour recovery period. Both CHO and CHO-P were superior to PLA (electrolytes and water) for supporting short-term recovery in masters class endurance athletes. The other main finding of this investigation was that both CHO and CHO-P appeared to be equally effective and significantly better than PLA in promoting HRR as assessed via the HRRi.

A similar study with younger participants [18] demonstrated an improvement in TTE performance (225.54 ± 20.12 sec) during a repeated bout of exercise following CHO-P (0.8 g/kg bm CHO + 0.4 g/kg bm PRO) consumption. Consumption of the CHO (1.2 g/kg bm) beverage attenuated the decrement in subsequent TTE performance (137.41 ± 18.90 sec) but was not significantly different from the PLA group (111.37 ± 22.01). In the current study, TTE performance in the second exercise bout improved for the CHO (295.48 ± 24.90 sec) and CHO-P (255.08 ± 25.07 sec) groups. The water and electrolyte solution was not sufficient for restoring TTE performance in the PLA group (171.13 ± 23.71 sec). Therefore, in the current study, the consumption of a CHO and CHO-P beverage improved TTE performance by 30% and 19%, respectively, whereas TTE performance declined by 20% in the PLA condition.

On an individual level, unadjusted values indicated 63% (5 of 8) of participants in the PLA group demonstrated a decline in TTE performance averaging a − 68 sec change from the first to second bout of exercise, with a range of −16 to −185 sec. In comparison, only one of seven participants in the CHO group experienced a decrease in TTE performance (−18-sec) during the subsequent exercise bout, with the range of *improvement* between 48 and 190 sec among all participants. Results were similar for the CHO-P group where one participant cycled 74 sec in both the first and second bouts of exercise, and all other performance times *increased* between 27 and 101 sec. The individual participant response is valuable for understanding the potential translation of findings from the laboratory to the field [40].

The MCAs who completed this study identified with a specific sport (i.e. primarily cycling but also running), and while as a group cannot be considered professional road cyclists, time-trial and exercise capacity testing are considered valid performance measurements for endurance athletes who regularly engage in prolonged high-intensity exercise that require periods of sustained power output [40]. In contrast, the heterogeneous sample of young college-aged adults in our previously published research [18] participated in numerous modes of exercise at a moderate-to-vigorous intensity [18]. Therefore, the homogeneous sample of MCAs actively engaged in an endurance sport that involves consistent training with periods of sustained power output may partly explain the difference in results such that in the current investigation both CHO and CHO-P treatments promoted an increase in TTE performance.

Three previously published studies that have examined post-exercise isocaloric CHO (1.2 g/kg bm) and CHO-P (0.8 g/kg bm CHO + 0.4 g/kg PRO) drinks and recovery from exhaustive exercise in young endurance-trained males have demonstrated that glycogen resynthesis postexercise is not necessarily the primary determinant of endurance-based performance outcomes [10,11,41]. The experimental protocol used in the present study was adapted from McCarthy and Spriet (2020), who estimated muscle glycogen depletion to be between approximately 55% to 65% following the first bout of high-intensity aerobic intervals and time to exhaustion. While glycogen synthesis was not measured in the present study, results were similar to the performance outcomes reported by Berardi et al. (2006) and Alghannam et al. (2016) [10,41]. Therefore, it is possible that the inclusion of protein in a recovery drink supports recovery in an important physiologic manner other than glycogen synthesis.

TTE at 90% PPO, while significantly improved during a subsequent bout of exercise relative to PLA, was not different between the CHO and CHO-P groups. CHO-P was not superior to CHO for improving acute TTE performance during a subsequent bout of high-intensity exercise; however, the addition of protein to a recovery beverage may have longer-term benefits. Doering et al. (2016) reported that a group of masters triathletes consumed 0.3 ± 0.2 g/kg bm of protein in the post-exercise recovery meal, which is slightly less than the recommended amount of 0.4 g/kg bm that has been found to maximally stimulate muscle protein synthesis in older adults [25]. In support, Churchward-Venne et al. (2020) reported that 30 g of protein (0.49 g protein/kg) co-ingested with 45 g of carbohydrate was found to maximally stimulate rates of myofibrillar protein synthesis in young adult males (27 ± 1 years) following 90 min of continuous cycling at 60% of maximal workload capacity. In the current study, the addition of whey protein to the CHO-P recovery beverage in the amount of 0.4 g/kg bm was approximately 32 ± 2.5 g, which is in line with current recommendations for post-exercise nutrient intake [25,42].

We found that adding protein to the recovery drink did not interfere with the acute performance benefits also seen in the CHO group. The inclusion of protein in the recovery drink has additional benefits, such as creating a more anabolic environment. As a result, athletes may be able to adjust more quickly to changes in their training schedules when they add protein to their diet. In support, Churchward-Venne et al. (2020) reported that whole-body net protein balance was *negative* when participants consumed a CHO-only beverage (45 g). A *negative* nitrogen balance with a CHO-only beverage was also a primary finding for both Rustad et al. (2016) and Dahl et al. (2020) when endurance-trained participants consumed energy-matched recovery drinks and performed repeated bouts of high-intensity exercise and time to exhaustion cycling. Conversely, consumption of the CHO-P beverage resulted in a *positive* nitrogen balance in addition to improved TTE performance [11,43].

The current investigation utilized a novel index to examine HRR relative to total work. Results indicated an improved recovery for both the CHO and CHO-P conditions at each of the three time points postexercise, with no difference between conditions. In comparison, the PLA condition was inadequate for promoting HRR at 1-, 2-, and 5-min postexercise as evidenced by a significantly higher HRRi value. The findings of an improved recovery for the CHO and CHO-P conditions are similar to the findings of Moreno and colleagues (2013) and others [32]. In the study of Moreno et al. (2012), consumption of 1.4 ± 0.5 L of an isotonic solution (i.e. Gatorade®) during 90 min of exercise at 60% VO_2_peak and throughout the 60 min recovery period enhanced recovery of parasympathetic activity by 25 min of exercise cessation [30]. In the current study, the average change in body mass resulting from fluid loss during the first bout of aerobic intervals and TTE for all participants was 0.7 ± 0.2 kg. Weight either approached or returned to baseline for all participants following consumption of 1.1 ± 0.2 liters of fluid during the 2-hour recovery period. In support, Moreno et al. (2013) reported lack of fluid intake during exercise and recovery resulted in approximately 2.0 ± 0.6% loss of body weight, as compared to −0.2 ± 0.7% with the recovery beverage. Consequently, indices of vagal activity as assessed by heart rate variability did not fully recover by the end of the 60-min recovery period when participants received no fluid intake [30].

Even though in the current study PLA provided similar amounts of water and electrolytes, it is possible that HRR may not be a suitable indicator for assessing recovery of parasympathetic activity either with fluid alone (i.e. water), within the first 5 min of exercise cessation, or following high-intensity exercise [29,31]. Additionally, a delay in parasympathetic reactivation [44] and sympathetic withdrawal [45] during the first 10 min of recovery from high-intensity exercise has been demonstrated following repeated sprints (15-m sprints with 17s passive recovery) and continuous exercise at 90%-95% of heart rate reserve. It is possible that HRR becomes a more sensitive indicator when calculated relative to total work (HRRi) and when the HRRi is used in conjunction with high-intensity exercise, though additional research is warranted to support these findings. Therefore, the high-intensity aerobic interval and TTE protocol (20 min at 70–80% PPO plus TTE at 90% of PPO) employed in this study may necessitate caloric intake in the form of rapidly digestible CHO or CHO-P intake during a 2-hour recovery period to maximize HRR post exhaustive exercise, or the postexercise recovery period may need to extend beyond 5 min to fully evaluate HRR from exhaustive exercise.

There are several limitations to this study, including the rate of compliance completing the electronic 24-hour dietary assessment. In addition, dietary intake was not standardized among participants prior to the experimental protocol; however, statistical analysis revealed no significant differences in energy intake or macronutrient distribution between the three treatment groups for those athletes that did comply. We did not standardize dietary intake on the day of the experimental protocol nor was the high-intensity exercise examined in a fasted state. We also did not quantify or restrict caffeine intake 24 hours prior to testing. The goal of this investigation was to examine recovery from high-intensity exercise in an applied manner which allowed for exercise testing without a drastic change to the participant’s habitual diet or nutritional pre-exercise practice [40]. Alternatively, participants were instructed to keep their dietary intake consistent on both the day of familiarization and the experimental protocol. While all participants were instructed to consume the recovery drink in its entirety within 90 min and were monitored to ensure compliance, individual and group completion times are not provided. Due to the exhaustive nature of the high-intensity exercise protocol employed in this study, many participants chose to terminate the prescribed 5-min cool down period early. Future research using the high-intensity aerobic interval and TTE protocol employed in this study may consider a standardized cool down following TTE testing (i.e. mandatory 3–5 min of self-paced cycling without resistance) followed by 15–30 min in the seated or supine position for assessment of HRR.

This is the first study to examine the effect of an isocaloric CHO vs CHO-P drink on performance during a repeated bout of exhaustive exercise following a 2-hour recovery period in MCAs. The primary novel outcome of this study was that CHO-P was equally effective as CHO for enhancing performance in a subsequent bout of high-intensity exercise with a limited time to recover. In addition, the incorporation of a novel index to examine HRR relative to total work (HRRi) indicated an improved recovery for both the CHO-P and CHO conditions within the first 5 min of exercise cessation, in comparison to a water and electrolyte solution. This study found that the use of an adequate CHO-containing nutritional beverage significantly improved recovery in the group of masters class endurance athletes, while replacing a portion of the carbohydrate content with protein had no adverse effects on either recovery or performance.
